# Image Quality and Radiation Dose of CT Coronary Angiography with Automatic Tube Current Modulation and Strong Adaptive Iterative Dose Reduction Three-Dimensional (AIDR3D)

**DOI:** 10.1371/journal.pone.0142185

**Published:** 2015-11-23

**Authors:** Hesong Shen, Guochao Dai, Mingyue Luo, Chaijie Duan, Wenli Cai, Dan Liang, Xinhua Wang, Dongyun Zhu, Wenru Li, Jianping Qiu

**Affiliations:** 1 Department of Radiology, The Sixth Affiliated Hospital of Sun Yat-sen University, Guangzhou, Guangdong, China; 2 Department of Radiology, Chongqing Cancer Institute, Chongqing, China; 3 Imaging Center, The First People's Hospital of Kashi Area, Xinjiang, China; 4 Research Center of Biomedical Engineering, Graduate School at Shenzhen, Tsinghua University, Shenzhen, Guangdong, China; 5 Department of Radiology, Massachusetts General Hospital and Harvard Medical School, Boston, Massachusetts, United States; North Shore Long Island Jewish Health System, UNITED STATES

## Abstract

**Purpose:**

To investigate image quality and radiation dose of CT coronary angiography (CTCA) scanned using automatic tube current modulation (ATCM) and reconstructed by strong adaptive iterative dose reduction three-dimensional (AIDR3D).

**Methods:**

Eighty-four consecutive CTCA patients were collected for the study. All patients were scanned using ATCM and reconstructed with strong AIDR3D, standard AIDR3D and filtered back-projection (FBP) respectively. Two radiologists who were blinded to the patients' clinical data and reconstruction methods evaluated image quality. Quantitative image quality evaluation included image noise, signal-to-noise ratio (SNR), and contrast-to-noise ratio (CNR). To evaluate image quality qualitatively, coronary artery is classified into 15 segments based on the modified guidelines of the American Heart Association. Qualitative image quality was evaluated using a 4-point scale. Radiation dose was calculated based on dose-length product.

**Results:**

Compared with standard AIDR3D, strong AIDR3D had lower image noise, higher SNR and CNR, their differences were all statistically significant (*P*<0.05); compared with FBP, strong AIDR3D decreased image noise by 46.1%, increased SNR by 84.7%, and improved CNR by 82.2%, their differences were all statistically significant (*P*<0.05 or 0.001). Segments with diagnostic image quality for strong AIDR3D were 336 (100.0%), 486 (96.4%), and 394 (93.8%) in proximal, middle, and distal part respectively; whereas those for standard AIDR3D were 332 (98.8%), 472 (93.7%), 378 (90.0%), respectively; those for FBP were 217 (64.6%), 173 (34.3%), 114 (27.1%), respectively; total segments with diagnostic image quality in strong AIDR3D (1216, 96.5%) were higher than those of standard AIDR3D (1182, 93.8%) and FBP (504, 40.0%); the differences between strong AIDR3D and standard AIDR3D, strong AIDR3D and FBP were all statistically significant (*P*<0.05 or 0.001). The mean effective radiation dose was (2.55±1.21) mSv.

**Conclusion:**

Compared with standard AIDR3D and FBP, CTCA with ATCM and strong AIDR3D could significantly improve both quantitative and qualitative image quality.

## Introduction

CT coronary angiography (CTCA) has become the primary noninvasive imaging modality that enables accurate diagnosis or exclusion of coronary artery disease. However, CTCA usually exposes the patient to some ionizing radiation, and increasing healthcare concerns have been raised regarding its hazard [1–2]. At present, radiation dose using the mainstream 64-slice CTCA in routine clinical practice was reported to be as high as 8–18 mSv [3]. To reduce the radiation dose in CTCA imaging while maintaining diagnostic image quality, a number of approaches were developed including prospective electrocardiogram-triggered acquisition, heart rate reduction, denoising, high-pitch helical scanning, minimized z-axis scan range, tube voltage reduction, electrocardiogram-based tube current modulation, automatic tube current modulation (ATCM), and new iterative reconstruction methods [4–7].

ATCM selects the optimal tube current in an automated manner by using the attenuation values on anteroposterior and lateral scanogram. Adaptive iterative dose reduction three-dimensional (AIDR3D) is an iterative reconstruction method developed by Toshiba Medical Systems, which incorporates the statistical and scanner models for projection data, and multiple cycles of iteration for noise reduction until the desired noise level is achieved [8–11]. AIDR3D is expected to overcome the intrinsic limitations of conventional filtered back-projection (FBP) reconstruction, reduce image noise and improve image quality [12–14]. AIDR3D has three predetermined strength modes: mild, standard and strong, which define different blending ratio of AIDR3D and FBP in the iterative reconstruction process.

The combination of ATCM with AIDR3D may potentially further reduce radiation dose while achieving diagnostic image quality. To the best of our knowledge, there is only one study that has investigated the image quality of CTCA with automatic exposure control system and standard AIDR3D [9], no previous study evaluated the effect of CTCA with ATCM and strong AIDR3D. Study of Feger [15] with 320-slice CTCA showed that, strong AIDR3D had the lowest noise, the best signal-to-noise ratio (SNR) and contrast-to-noise ratio (CNR), compared with standard AIDR3D, mild AIDR3D, and FBP with quantum denoising software. Thus, the purpose of our study was to prospectively investigate image quality and radiation dose of CTCA acquired with ATCM and reconstructed by strong AIDR3D by comparison with those reconstructed by standard AIDR3D and FBP.

## Materials and Methods

### Ethics statement

The prospective study received ethical approval from Ethics Committee of the Sixth Affiliated Hospital of Sun Yat-sen University in China. The possible adverse effects of iodinated contrast medium injection and radiation exposure as well as the complications of CTCA were explained to all patients. Written informed consent was obtained from each patient to participate in this study.

### Clinical data

A total of 84 consecutive patients, who were clinically scheduled for cardiac CT for evaluation of coronary artery disease, were prospectively recruited in this study. Exclusion criteria were a history of iodinated contrast medium allergic reaction, impaired renal function, previous coronary artery interventions including stenting and/or coronary artery bypass grafts, as well as those with heart rates more than 65 beats per minute after beta-blocker premedication. The patient demographics were described in Table 1.

**Table 1:** Patient demographics.

**Gender (Male: Female)**	57: 27
**Age (Years old)**	58.0 ± 17.3 (30–80)
**Body weight (Kg)**	60.3 ± 13.7 (51–78)
**Body mass index (Kgm** ^**-2**^ **)**	21.1 ± 3.2 (17–27)
**Heart rate at CT scan (Beat per minute)**	58.4 ± 6.2 (52–65)
**Risk factors of coronary artery disease**	84
**Chest pain**	52
**Dyspnea**	13
**Abnormal result of electrocardiogram**	9
**Abnormal result of cardiac echo**	6
**Abnormal result of treadmill test**	4

### CT image data acquisition and reconstruction

Three minutes before CT scanning, 0.5 mg of sublingual nitroglycerin was administered for all patients without contraindication. A total of 60 mL of nonionic contrast medium (Ultravist, 370 mgI/ml; Schering Pharmacy, Guangzhou, China) was injected into the antecubital vein at 6 mL/s, followed by 20 mL of 0.9% normal saline at 5 mL/s, by using a dual power injector. CT image data were acquired using a 640-slice dynamic volume CT scanner (Aquilion ONE TSX-30A; Toshiba Medical Systems, Tochiki-ken, Japan). A single gantry rotation occurred for all image data acquisitions in moderate inspiration with breath-holding. Mid-diastolic prospective scanning with an electrocardiogram-gated window of 75% of the R-R interval was performed. ATCM (^SURE^Exposure; Toshiba Medical Systems, Tochiki-ken, Japan) was employed, with gantry rotation time of 350 ms and tube voltage fixed at 120 kVp. The level of image noise was predetermined at a standard deviation of 45 (0.5 mm section thickness, normal soft tissue reconstruction kernel being FC43). A medium field of view of the maximal 140 mm in the z-axis was selected. CT acquisition was automatically triggered when a signal intensity of contrast medium threshold of 340 HU was reached in the left ventricle.

The original cross-sectional CT images were reconstructed with strong AIDR3D (strong level of blending, the computational time is 30 reconstructed images per second), standard AIDR3D and FBP. Reconstruction thickness was 0.5 mm, and reconstruction interval was 0.25 mm.

### CTCA images

The strong AIDR3D, standard AIDR3D and FBP reconstructed image data were transferred to an image post-processing workstation (Vitrea, Version 6.2; Toshiba Medical System, Tochiki-ken, Japan) via picture archiving and communication system (PACS). CTCA images were obtained with curved planar reformation in the image post-processing workstation.

### Quantitative image quality evaluation

Two experienced cardiovascular radiologists, who were blinded to the patients' clinical data and the reconstruction methods (strong AIDR3D, standard AIDR3D or FBP) jointly analyzed the CTCA images by measuring image noises, SNRs, and CNRs. In case of discrepancy happens, the two radiologists reached a consensus after reviewing and discussing the controversial images with a senior radiologist. CT density was measured at aortic root cranial to left coronary ostium, left main coronary artery, and epicardial fat surrounding left main coronary artery. Each region of interest was drawn to be as large as possible with particular cautions to exclude calcifications, plaques, stenoses and vessel wall. Image noise was defined as the standard deviation of CT density at aortic root cranial to left coronary ostium. SNR was calculated by dividing the CT density of left main coronary artery by image noise. As for CNR, CT density of left main coronary artery was first subtracted by that of epicardial fat surrounding left main coronary artery and then divided by image noise [4, 5, 16].

### Qualitative image quality evaluation

The aforementioned two radiologists performed the qualitative evaluation in the same blinded manner. According to the modified guidelines of the American Heart Association, coronary artery is classified into 15 segments. The 15 coronary artery segments are further categorized into three parts: (1) proximal part (proximal right coronary artery, left main coronary artery, proximal left circumflex artery, proximal left anterior descending artery), (2) middle part (middle right coronary artery, distal right coronary artery, ramus intermedius, obtuse marginalis, first diagonal branch, middle left anterior descending artery), and (3) distal part (posterior descending artery, distal left circumflex artery, second diagonal branch, distal left anterior descending artery). Qualitative CTCA image quality was evaluated using a 4-point scale [17]: 4 = excellence (excellent attenuation of vessel lumen and clear vessel wall definition with barely perceived image noise), 3 = good (good attenuation of vessel lumen and well-maintained vessel wall definition with minimal image noise), 2 = fair (some limitations in vessel wall definition and contrast resolution with moderate image noise that would affect diagnosis), and 1 = poor (impaired image quality with severe image noise). Mean scores were calculated for three parts of coronary artery segments. Images with a qualitative quality score of 4 or 3 could be used for diagnosis, whereas images with a qualitative quality score of 2 or 1 could not be used for diagnostic purpose.

### Calculation of radiation dose

Dose-length product in each scanning reported by the CT scanner was recorded. Radiation dose was calculated by the effective radiation dose: (dose-length product) × (conversion factor), where the conversion factor varies at different body parts. The conversion factor value specific to cardiac body part is 0.017 [18].

### Statistical analysis

Continuous variables were presented as mean ± standard deviation. All statistical analyses were performed using SPSS for Windows version 16.0. A paired, two-tailed Student’s *t* test was applied to assess whether there was a statistically significant difference measurements data between strong AIDR3D, standard AIDR3D and FBP. Inter-observer agreement was performed with kappa statistic [19], which was interpreted as poor (kappa coefficient k = 0.00–0.20), fair (k = 0.21–0.40), moderate (k = 0.41–0.60), good (k = 0.61–0.80), excellent (k = 0.81–1.00). A *P* value of 0.05 or less was considered to be statistically significant.

## Results

### Quantitative image quality of CTCA

The results of quantitative image quality analysis were listed in Table 2. Compared with standard AIDR3D, strong AIDR3D had lower image noise, higher SNR and CNR, their differences were all statistically significant (*P*<0.05). Compared with FBP, strong AIDR3D decreased image noise by 46.1%, increased SNR by 84.7%, and improved CNR by 82.2%, their differences were all statistically significant (*P*<0.05 or 0.001).

**Table 2:** Quantitative image quality of CTCA with ATCM and strong AIDR3D, standard AIDR3D and FBP.

Item	Strong AIDR3D	Standard AIDR3D	FBP	*P1*	*P2*
**Noise (HU)**	27.2±4.4	33.3±5.2	60.0±12.4	<0.05	<0.001
**SNR**	21.1±5.1	17.1±3.1	11.4±2.8	<0.05	<0.05
**CNR**	24.7±5.1	19.1±2.9	13.5±3.2	<0.05	<0.001

Noise = Image noise; SNR = Signal-to-noise ratio; CNR = Contrast-to-noise ratio. *P1*: Strong AIDR3D compared with standard AIDR3D; *P2*: Strong AIDR3D compared with FBP.

### Qualitative image quality of CTCA

Inter-observer agreement for qualitative image quality evaluation was interpreted as excellent (k = 0.83, *P*<0.05).

**Fig 1:**
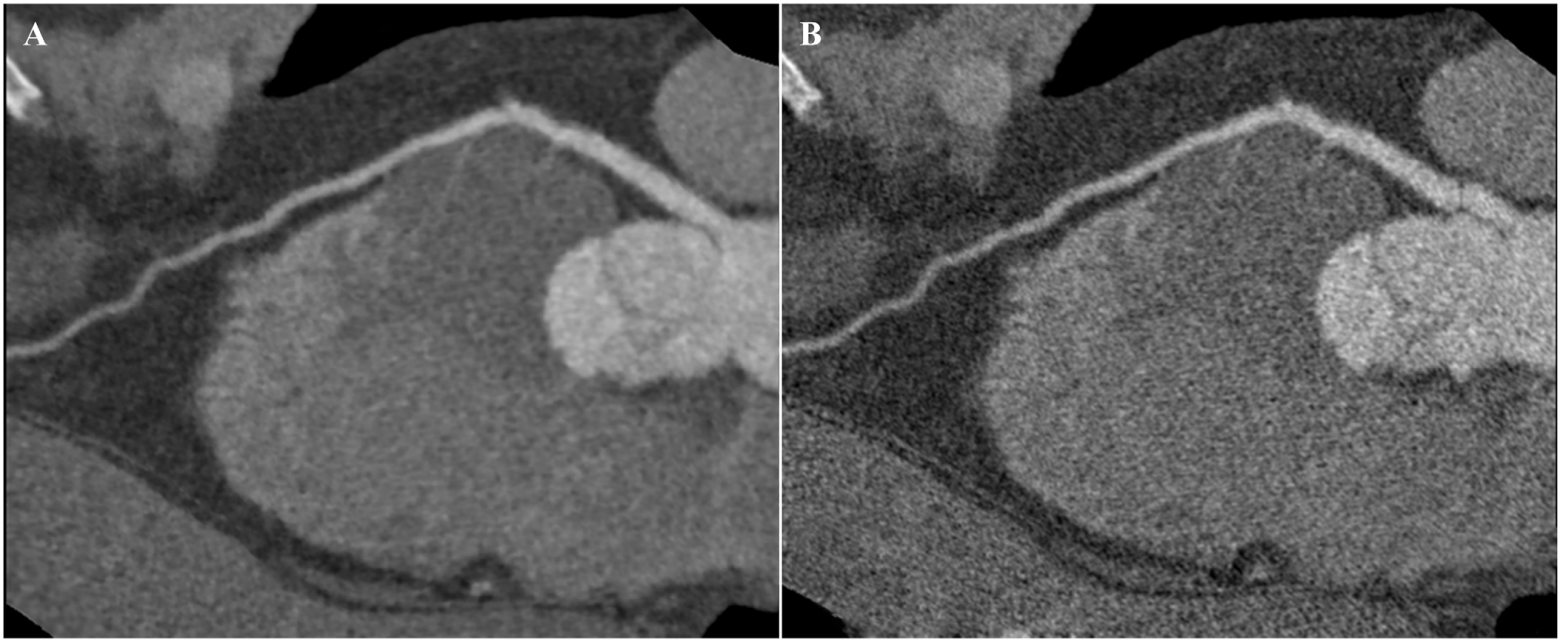
CTCA images of left anterior descending artery. A: Image with a qualitative quality score of 4 with ATCM and strong AIDR3D, excellent attenuation of vessel lumen and clear vessel wall definition with barely perceived image noise. B: Image with a qualitative quality score of 3 with ATCM and FBP, good attenuation of vessel lumen and well-maintained vessel wall definition with minimal image noise.

**Fig 2:**
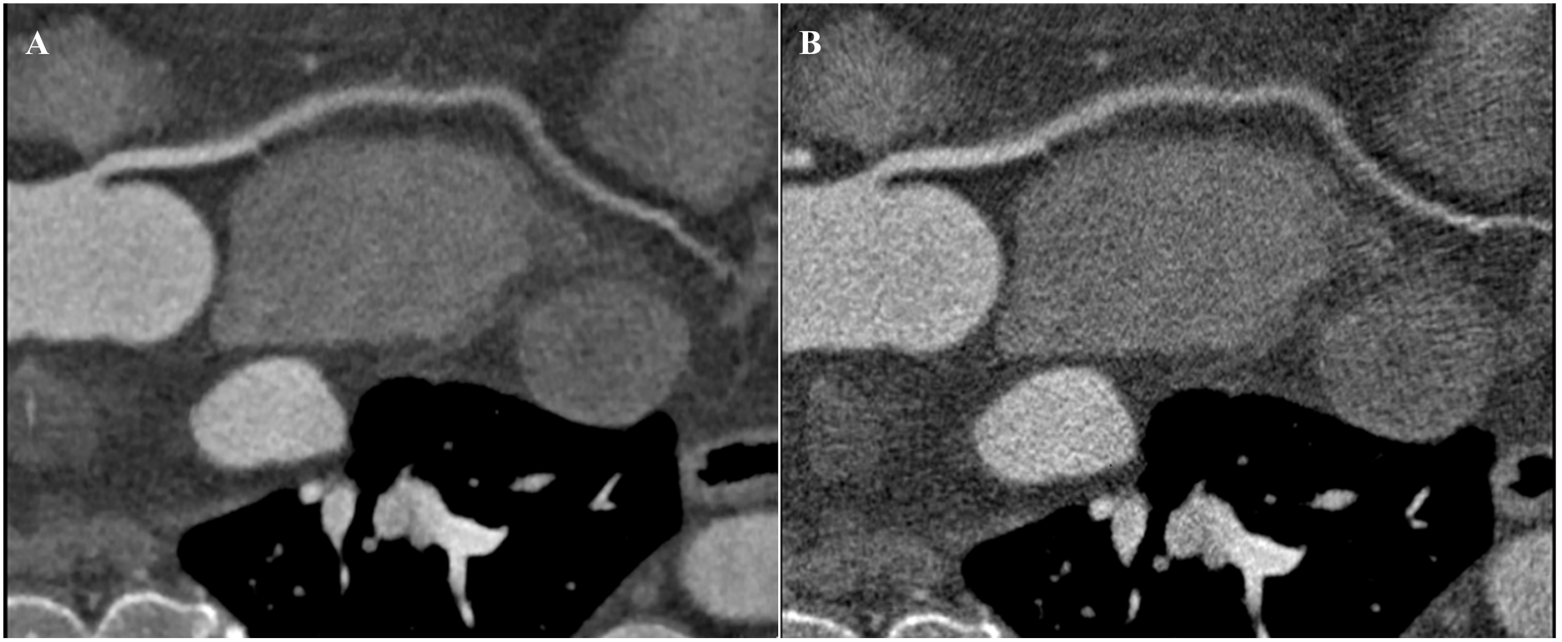
CTCA images of right coronary artery. A: Image with a qualitative quality score of 2 with ATCM and strong AIDR3D, some limitations in vessel wall definition and contrast resolution with moderate image noise that would affect diagnosis. B: Image with a qualitative quality score of 1 with ATCM and FBP, impaired image quality with severe image noise.

**Fig 3:**
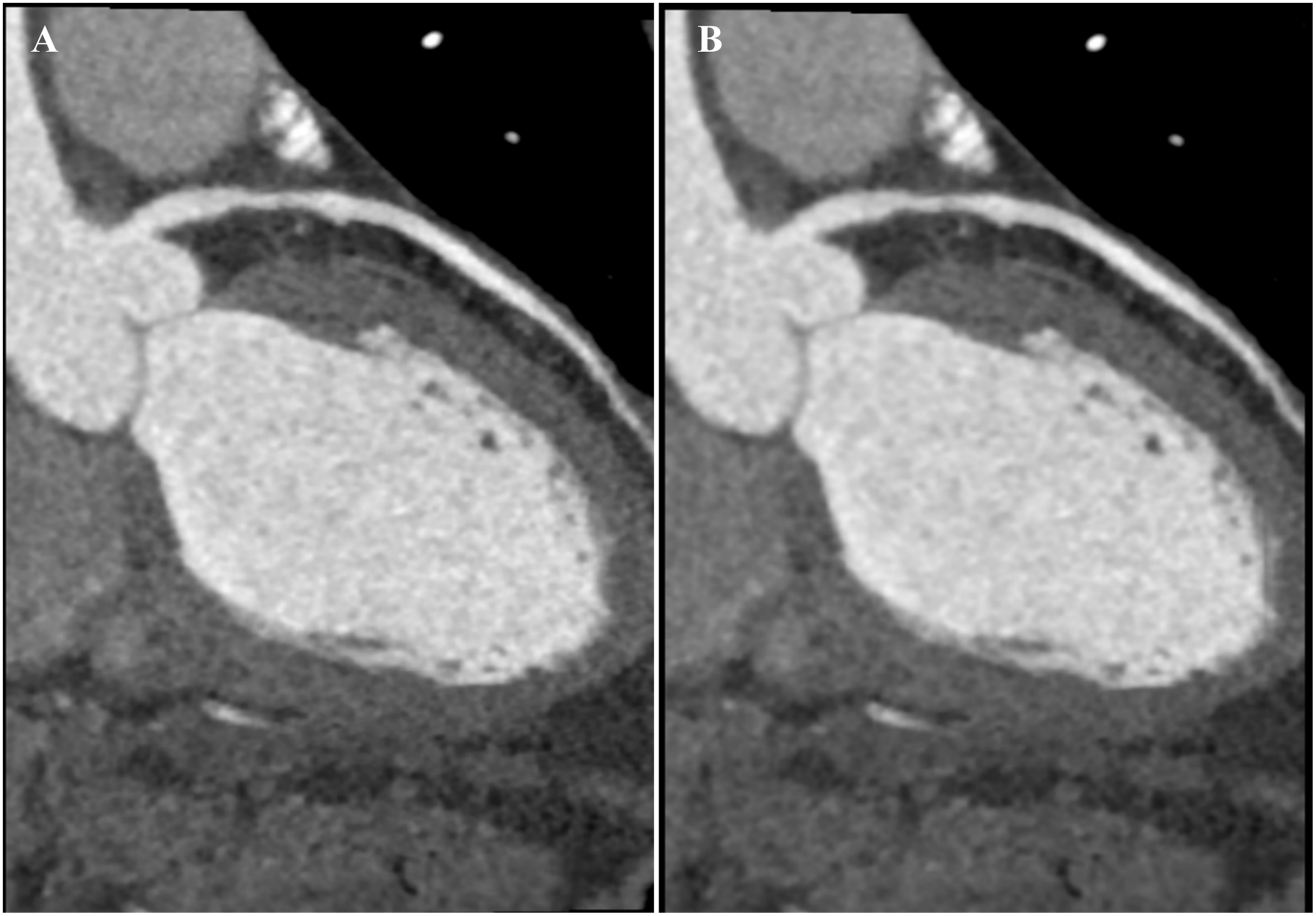
CTCA images of left anterior descending artery. A: Image with a qualitative quality score of 4 with ATCM and strong AIDR3D, excellent attenuation of vessel lumen and clear vessel wall definition with barely perceived image noise. B: Image with a qualitative quality score of 3 with ATCM and standard AIDR3D, good attenuation of vessel lumen and well-maintained vessel wall definition with minimal image noise.

**Fig 4:**
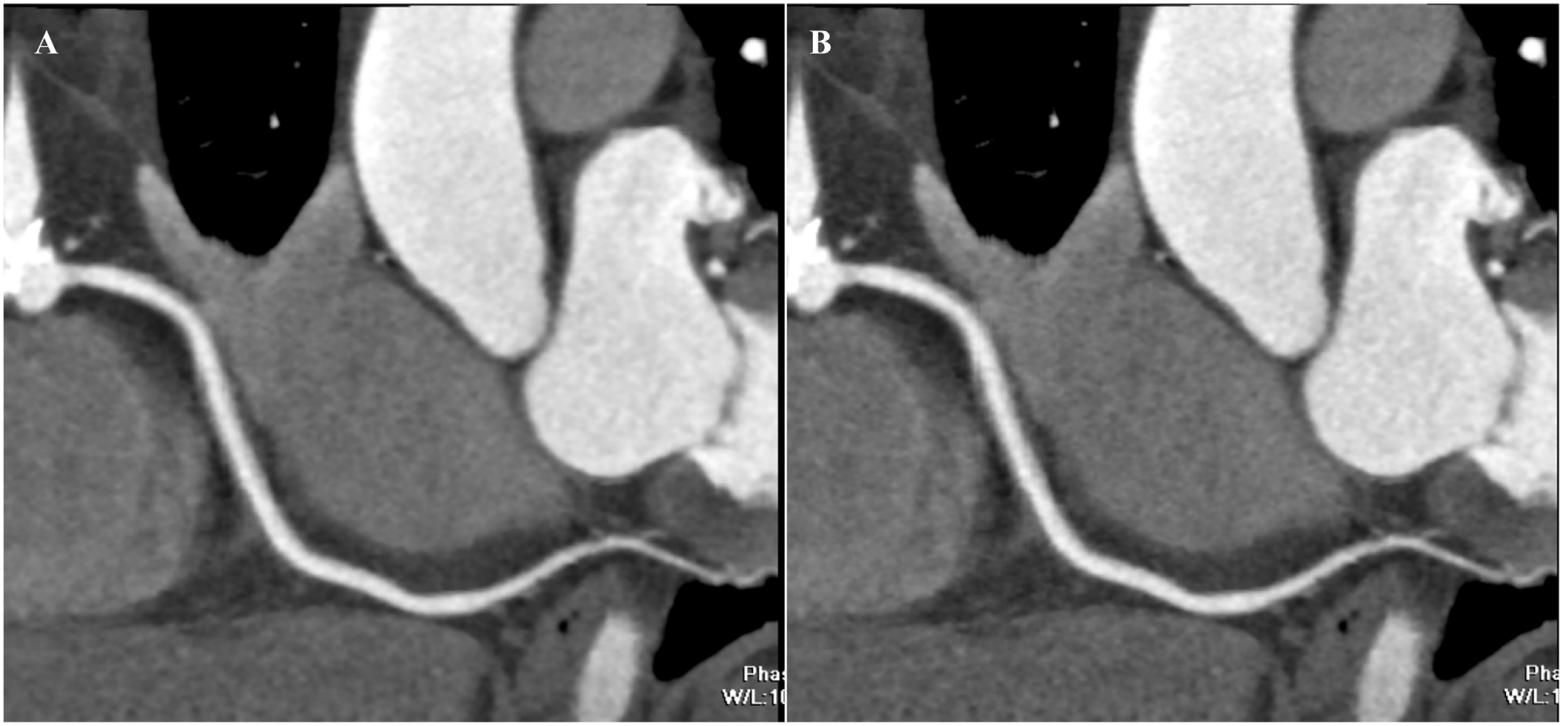
CTCA images of right coronary artery. A: Image with a qualitative quality score of 2 with ATCM and strong AIDR3D, some limitations in vessel wall definition and contrast resolution with moderate image noise that would affect diagnosis. B: Image with a qualitative quality score of 1 with ATCM and standard AIDR3D, impaired image quality with severe image noise.

There were in total of 1260 coronary artery segments in 84 patients including 336 proximal segments, 504 middle segments, and 420 distal segments. The 4-scale image qualities of three parts of segments were showed in Table 3. Segments with diagnostic image quality for strong AIDR3D were 336 (100.0%), 486 (96.4%), and 394 (93.8%) in proximal, middle and distal part respectively; whereas those for standard AIDR3D were 332 (98.8%), 472 (93.7%), 378 (90.0%), respectively; those for FBP were 217 (64.6%), 173 (34.3%), 114 (27.1%), respectively. Total segments with diagnostic image quality in strong AIDR3D (1216, 96.5%) were higher than those of standard AIDR3D (1182, 93.8%) and FBP (504, 40.0%). The differences between strong AIDR3D and standard AIDR3D, strong AIDR3D and FBP were all statistically significant (*P*<0.05 or 0.001) (Figs 1–4).

**Table 3:** Segments of qualitative image quality of three parts CTCA with ATCM and strong AIDR3D, standard AIDR3D and FBP.

	Proximal CTCA			Middle CTCA			Distal CTCA		
Score	Strong	Standard	FBP	Strong	Standard	FBP	Strong	Standard	FBP
**1**	0	1	12	0	3	47	2	4	75
**2**	0	3	107	18	29	284	24	38	231
**3**	39	38	213	102	99	173	99	95	112
**4**	297	294	4	384	373	0	295	283	2
**Total**	336	336	336	504	504	504	420	420	420

Strong = Strong AIDR3D; Standard = Standard AIDR3D.

### Radiation dose

Dose-length products reported by the CT scanner for 84 patients were from 64.29 mGy.cm to 471.43 mGy.cm, and the mean dose-length products was (150.00±71.43) mGy.cm. The effective radiation dose ranged from 1.29 mSv to 8.01 mSv. The mean effective radiation dose was (2.55±1.21) mSv.

## Discussion

FBP is a conventional CTCA image reconstruction method. However, applying FBP to low-dose CTCA may reduce the image quality because of quantum noises and electronic noises [20]. In this study, image noise, SNR and CNR in FBP reconstructed CTCA images were (60.0±12.4) HU, 11.4±2.8, 13.5±3.2, respectively. The total segments of diagnostic images were 504 (40.0%).

AIDR3D, the successor to AIDR, is the latest introduced three-dimensional adaptive iterative image reconstruction developed by Toshiba Medical System. It is designed to work using raw data and reconstruction process can adaptively balance the relationship between noise suppression and image details by repeated spatial filtering for noise suppression until a pre-defined target is reached, thereby improving image quality [5, 8, 10, 12, 13, 21, 22].

This is the first study to prospectively evaluate image quality and radiation dose of CTCA with ATCM and strong AIDR3D, compared with standard AIDR3D and FBP in 640-slice CT. In this study, image noise, SNR, and CNR of CTCA with strong AIDR3D were (27.2±4.4) HU, 21.1±5.1, 24.7±5.1, respectively; its mean effective radiation dose was (2.55±1.21) mSv. Compared with standard AIDR3D, strong AIDR3D not only had significantly lower image noise, significantly higher SNR and CNR, but also achieved significantly more diagnostic image quality in proximal, middle, distal part and total segments. Compared with FBP, strong AIDR3D decreased image noise by 46.1%, increased SNR by 84.7%, improved CNR by 82.2%, and had significantly more segments of diagnostic images.

Study of Tatsugami [23] with 320-slice CTCA showed that using of AIDR reduced image noise and improved image quality. Compared with FBP, AIDR would have a potential for further reduction of radiation dose in CTCA. The application of standard AIDR3D and individualized automatic tube current selection improved subjective image quality, and led to a 39% reduction in radiation dose. Tomizawa [24] found that, compared with FBP, 320-slice CTCA with AIDR has merit of an average of 40% reduction of tube current which resulted in 22% reduction in median radiation dose while maintaining subjective and objective image quality.

In Yoo [9] study, image noise, SNR and CNR of 640-slice CTCA with ATCM and standard AIDR3D were (45.0±9.4) HU, 15.0±2.1, 16.8±2.3, respectively. The mean effective radiation dose was the same as that in our study with ATCM and strong AIDR3D, but our study decreased image noise, increased SNR, and improved CNR. The mean effective radiation dose in our study is much lower than that of mainstream 64-slice CTCA in routine clinical practice (8–18 mSv) [3].

Protocol of Zhang [25] with prospectively electrocardiogram-triggered high-pitch coronary CT angiography at 70 kVp using 30 cc contrast medium resulted in an effective radiation dose of (0.2 ± 0.0) mSv and high diagnostic accuracy for stenosis detection, compared to invasive coronary angiography as reference standard, but it was in a selected and non-obese population. Study of Gordic [26] suggested that high-pitch coronary CT angiography with advanced modeled iterative reconstruction is possible at a radiation dose of (0.3 ± 0.1) mSv, however, it was also in a selected population. Zheng [27] found that combining iterative reconstruction techniques with prospectively electrocardiogram-triggered high-pitch spiral acquisition coronary CT angiography (2 × 128 × 0.6 mm, 300 mAs) and low-tube-voltage of 80 kVp, a low-concentration contrast medium of 270 mgI/mL could still maintain the contrast enhancement in coronary arteries without impairing image quality and at a significantly lower radiation dose of (0.26 ± 0.05) mSv, but it was only in fifty patients with body mass index ≤ 25 Kgm^-2^ and heart rate ≤ 65 beats per minute.

Our study has some limitations that should be taken into consideration. First, patient exclusion criteria included those with heart rates more than 65 beats per minute after beta-blocker premedication. This exclusion criterion may scan patients using a lower radiation dose than studies that include patients with higher heart rates. Second, we did not evaluate the diagnostic performance of strong AIDR3D. Subsequent studies assessing diagnostic performance of strong AIDR3D using conventional coronary angiography as the reference standard would be required to take a step further in establishing strong AIDR3D as the mainstream in routine practice. Third, we did not evaluate image quality with mild AIDR3D as Yoo [9] did in the study of 640-slice CTCA with standard AIDR3D. Fourth, because Yoo [9] have studied image quality of CTCA with automatic exposure control system and standard AIDR3D, we no longer compared quantitative and qualitative image quality between standard AIDR3D and FBP. Fifth, we did not use a lower tube voltage such as 100 kVp and 80 kVp, which may cause a higher degree of beam-hardening effects. Sixth, image quality of CTCA with strong AIDR3D enjoys less image noise, better SNR and CNR, but it has a potential limitation of over-smooth the image and lead to texture/structure change. Finally, we only evaluated an iterative reconstruction method developed by a single manufacturer. It is difficult to compare our results with other types of iterative reconstructions including Adaptive Statistical Iterative Reconstruction (ASIR, GE Healthcare), Model Based Iterative Reconstruction (MBIR, GE Healthcare), iDose (Philips Healthcare), Iterative Reconstruction in Image Space (IRIS, Siemens Healthcare), and Sinogram Affirmed Iterative Reconstruction (SAFIRE, Siemens Healthcare), as the individual implantation is different from each CT manufacturer.

In conclusion, our study suggested that compared with standard AIDR3D and FBP, CTCA with ATCM and strong AIDR3D could significantly improve both quantitative and qualitative image quality, its mean effective radiation dose was (2.55±1.21) mSv.
